# Supplementary material for: New Insights into Radio-Resistance Mechanism Revealed by (Phospho)Proteome Analysis of Deinococcus Radiodurans after Heavy Ion Irradiation
Source: Int J Mol Sci. 2023 Oct 1;24(19):14817. doi: 10.3390/ijms241914817 (PMC10572868; doi:10.3390/ijms241914817)

Contents

Supplemental figures

Figure S1. Venn diagram for identification of proteome and phosphoproteome at 0/2h after 0/20/80/160 (A-D) Gy irradiation.

Figure S2. Comparison of proteome and phosphoproteome identification results and quality control (A-C).

Figure S3. Analysis of differently expressed proteins at each irradiation dose and time point (A-F).

Figure S4. Analysis of differential phosphosites at each irradiation dose and time point (A-F).

Figure S5. A comparison of the upregulated (A) and downregulated (B) proteins between our proteome dataset and the published datasets.

Figure S6. Protein domain enrichment analysis of upregulated proteins in different time points.

Figure S7. Motif analysis of irradiation sensitive phosphopeptides (A-C).

Figure S8. Site disorder analysis.

Supplemental Tables

Table S1. Proteome(A) & phosphoproteome(B) identification and differential expression in *Deinococcus radiodurans*.

Table S1C. Comparison of the identification between the proteome and the phosphoproteome at each radiation states.

Table S1D. Comparison of the proteomics with previous work.

Table S2. GO entries, pathways, domains, gene age, disorder degree of all proteins in *Deinococcus radiodurans*.

Table S3A. The coverage and up-regulation ratio of (phospho)proteome identification of all pathways in *Deinococcus radiodurans*

Table S3B. Changes of proteins in oxidative phosphorylation pathway after irradiation

Table S4. Proteins contain kinase domains and their differential changes after irradiation.

Supplemental figure legends

Figure S1. Venn diagram for identification of proteome and phosphoproteome at 0/2h after 0/20/80/160 (A-D) Gy irradiation.

Figure S2. Comparison of proteome and phosphoproteome identification results and quality control (A-C).

(A, B) Violin diagram shows the comparison of expression levels between (phospho)proteome sample in each irradiation state. (C) The correlation between two experiment repeats of the proteome.

Figure S3. Analysis of differently expressed proteins at each irradiation dose and time point (A-F).

Volcanic plot to show the DEPs screened by $log_2FC > 1$, $FDR < 0.05$. 20 Gy, 80 Gy and 160 Gy were all compared with 0 Gy at the corresponding time point.

Figure S4. Analysis of differential phosphosites at each irradiation dose and time point (A-F).

Volcanic plot to show the differential phosphosites screened by $log_2FC > 1$, $FDR < 0.05$. 20 Gy, 80 Gy and 160 Gy were all compared with 0 Gy at the corresponding time point.

Figure S5. A comparison of the upregulated proteins between our proteome dataset and the published datasets.

Venn diagram shows comparison with other irradiation proteomics datasets, 740 upregulated proteins were identified only in this dataset.

Figure S6. Protein domain enrichment analysis of upregulated proteins in different time points.

The time points are distinguished by different colors, and the size of the circle in the

figure indicates the number of genes with that domain.

Figure S7. Motif analysis of irradiation sensitive phosphopeptides (A-C).

Analysis of motifs upstream and downstream of differential phosphosites. Distinguish between serine (S), threonine (T) and tyrosine (Y).

Figure S8. Site disorder analysis.

Whether the phosphosites and other AA sites are located in a disordered region. Phosphosites tend to be disordered, the difference is significant (fisher exact t test, P = 3.98e-84).

Figure S1. Venn diagram for identification of proteome and phosphoproteome at 0/2h after 0/20/80/160 (A-D) Gy irradiation.

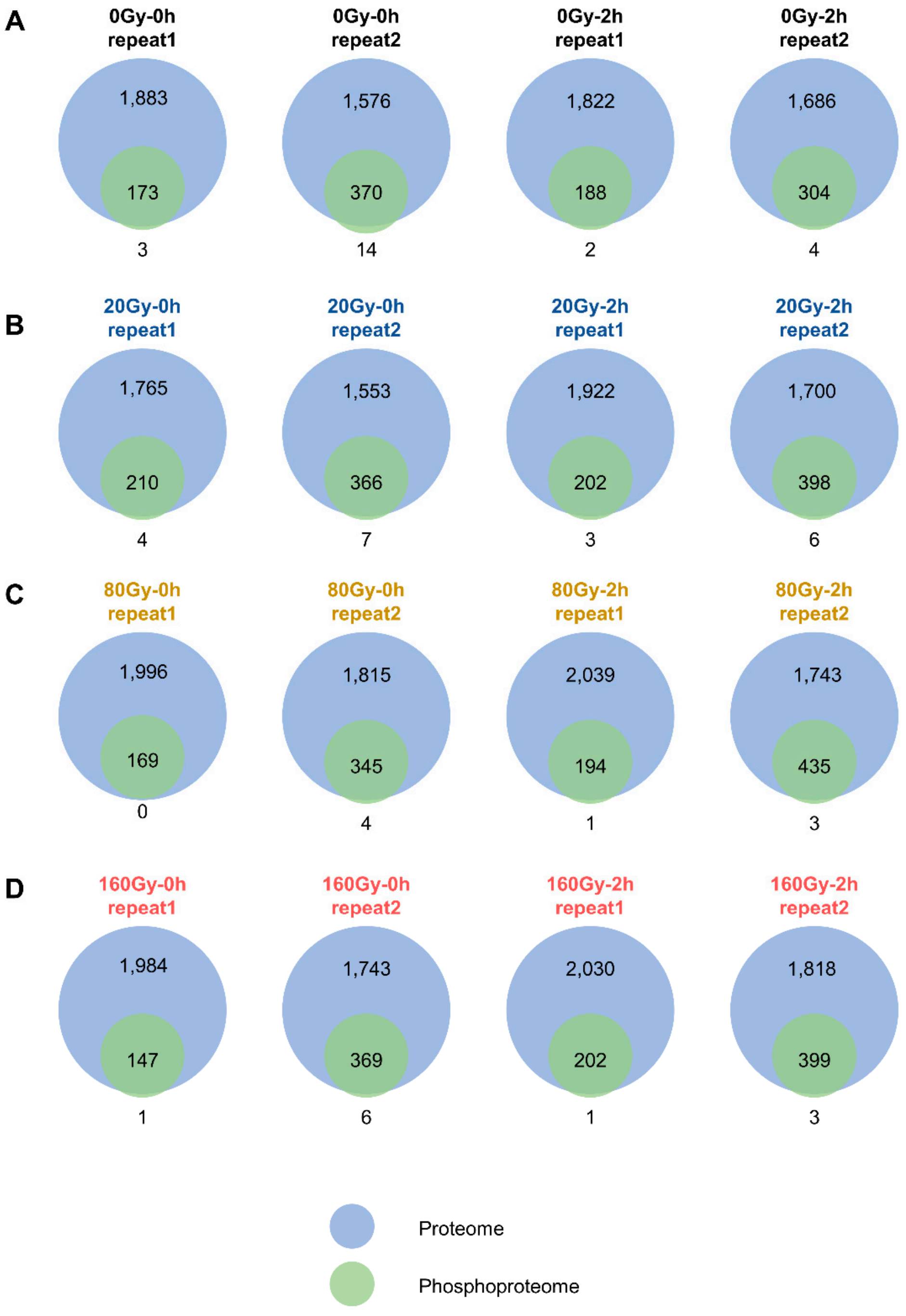

Figure S2. Comparison of proteome and phosphoproteome identification results and quality control (A-C).

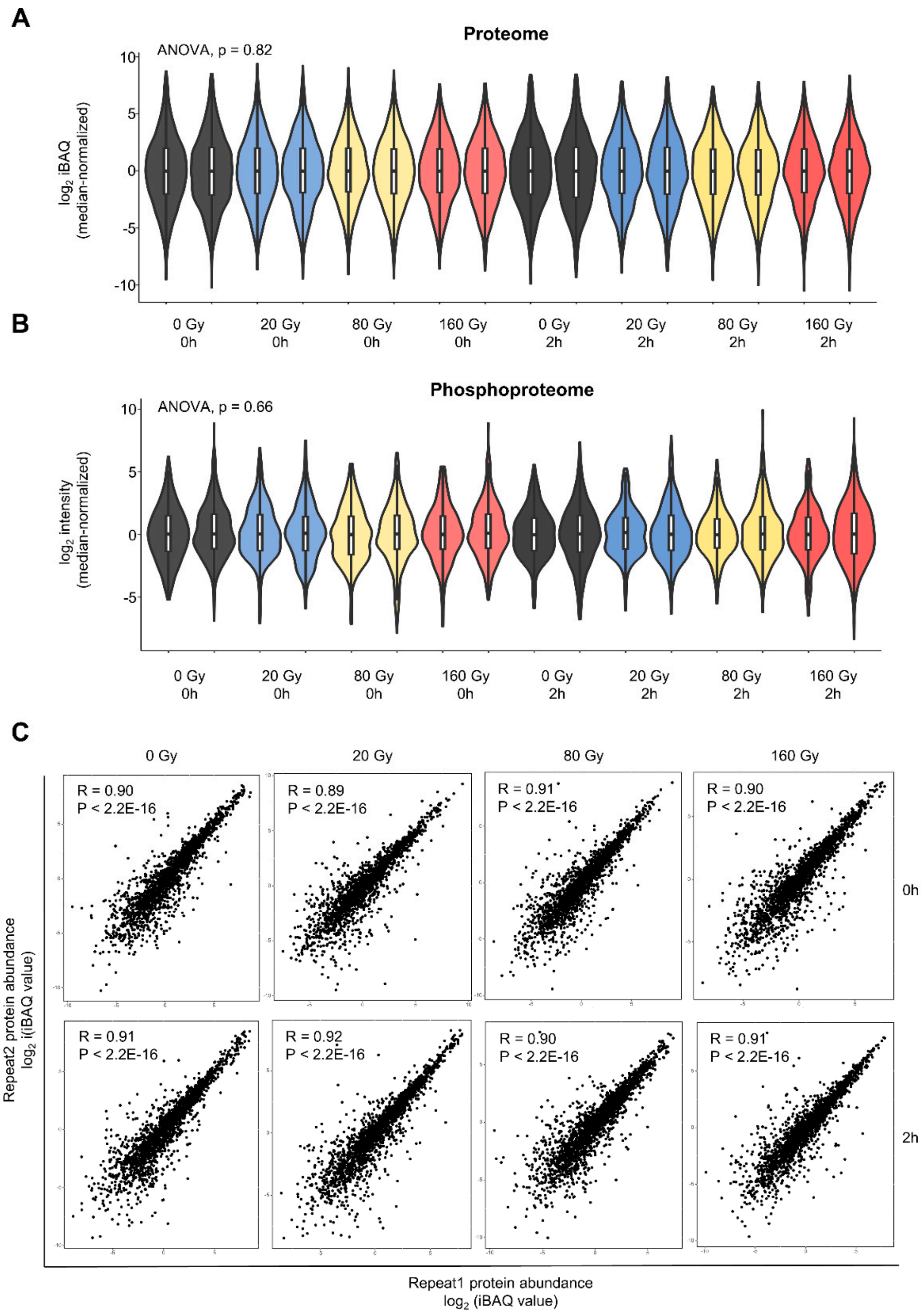

Figure S3. Analysis of differently expressed proteins at each irradiation dose and time point (A-F).

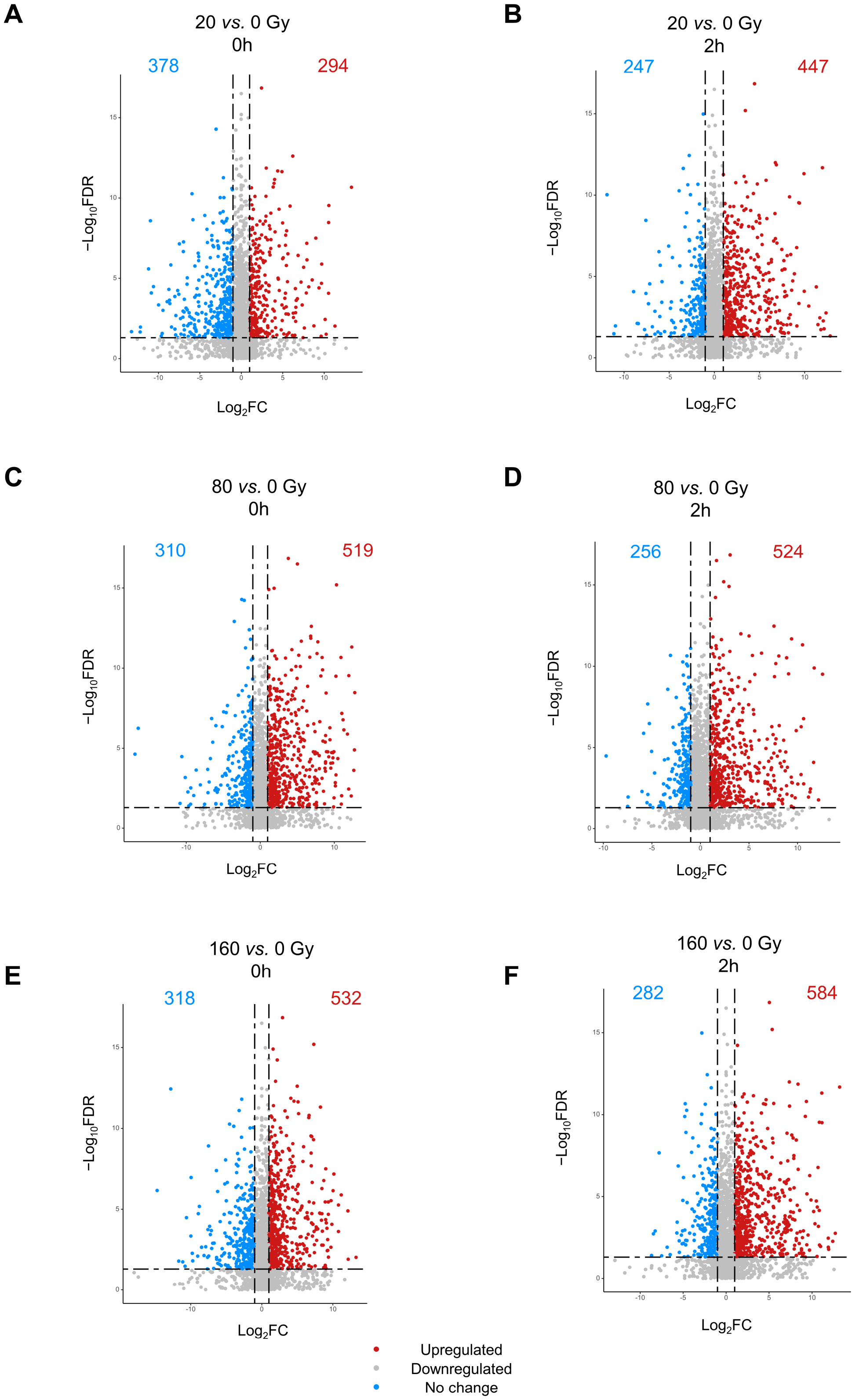

Figure S4. Analysis of differential phosphosites at each irradiation dose and time point (A-F).

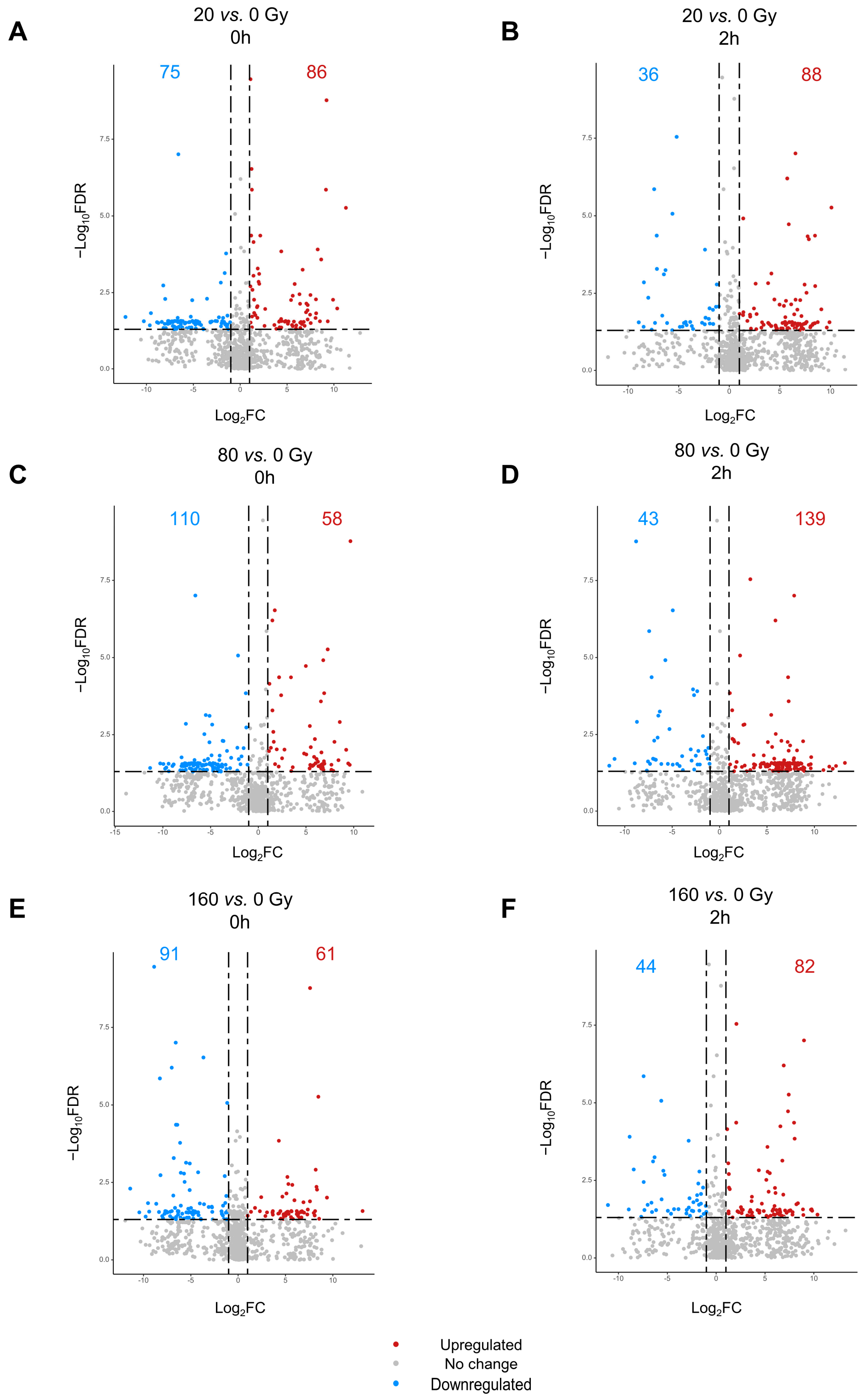

Figure S5. A comparison of the upregulated (A) and downregulated (B) proteins between our proteome dataset and the published datasets.

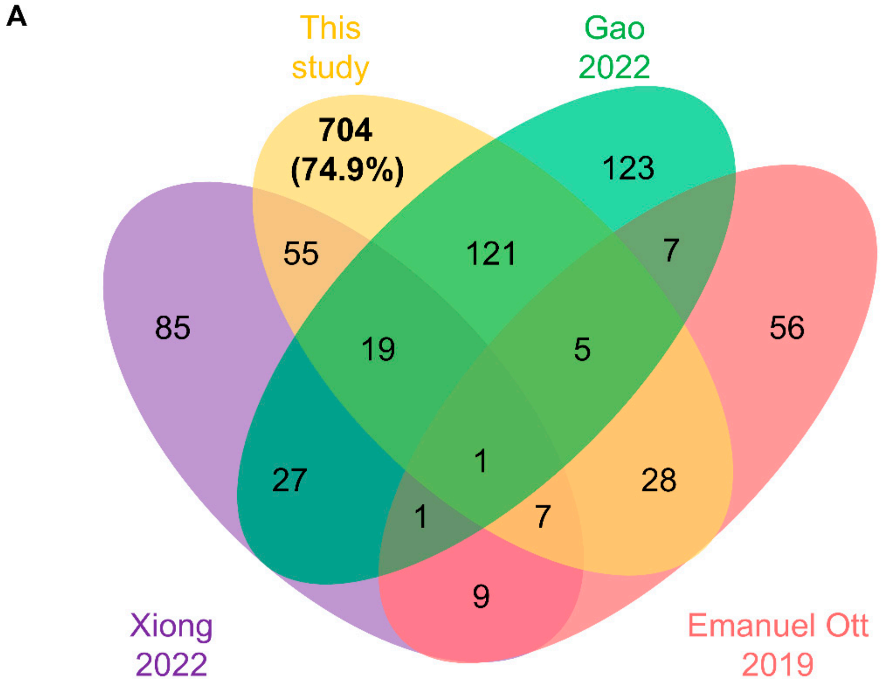

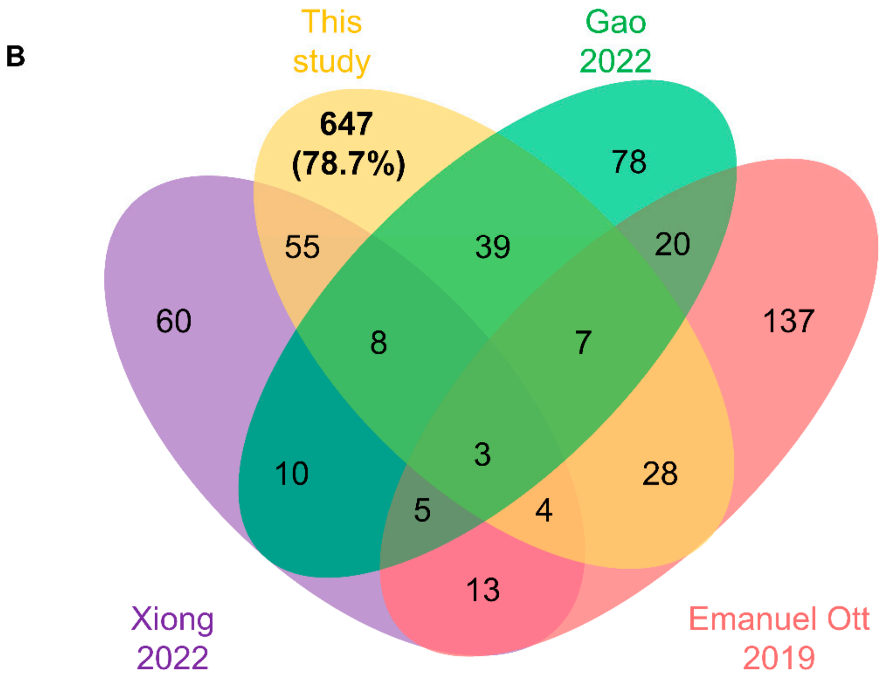

Figure S6. Protein domain enrichment analysis of upregulated proteins in different time points.

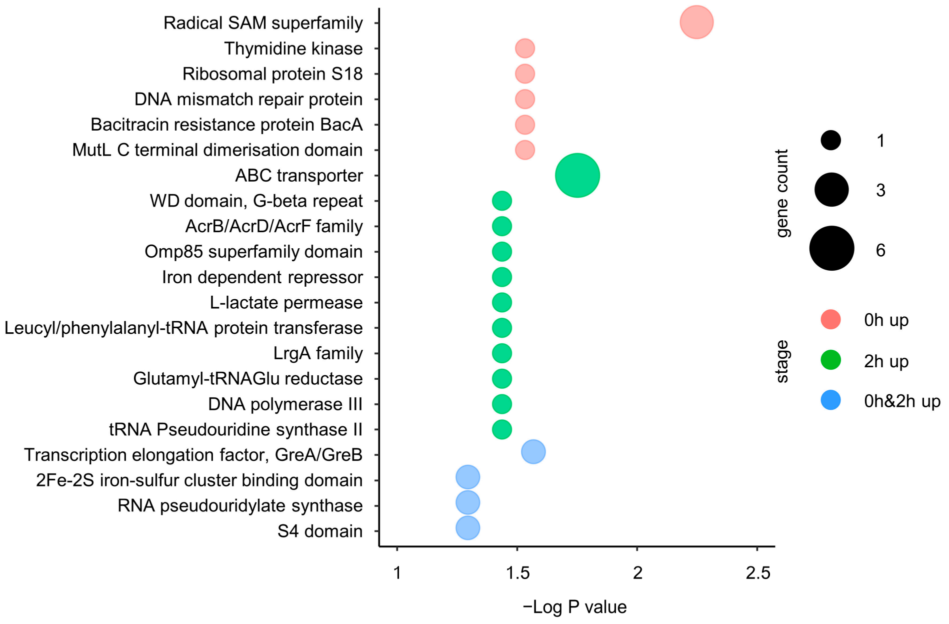

Figure S7. Motif analysis of irradiation sensitive phosphopeptides (A-C).

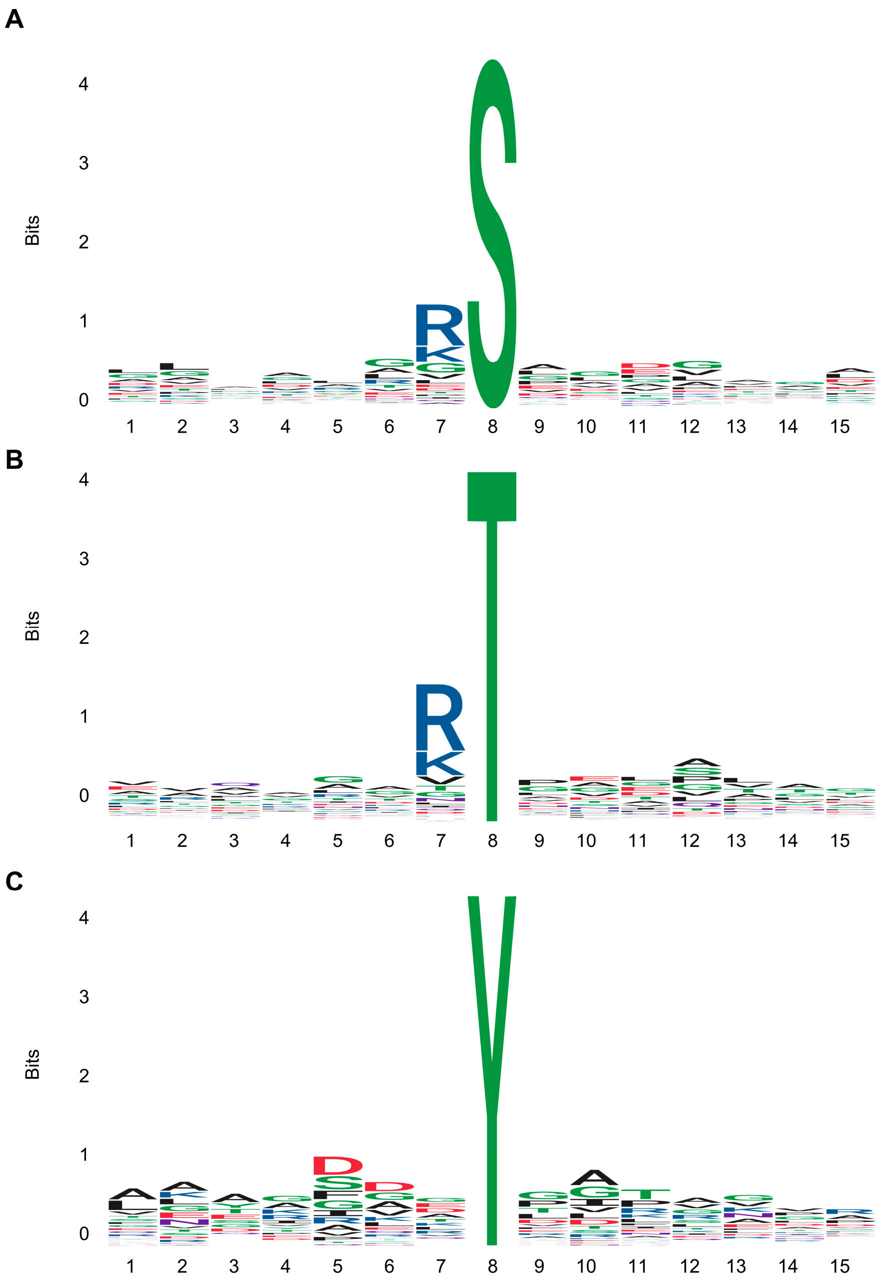

Figure S8. Site disorder analysis.

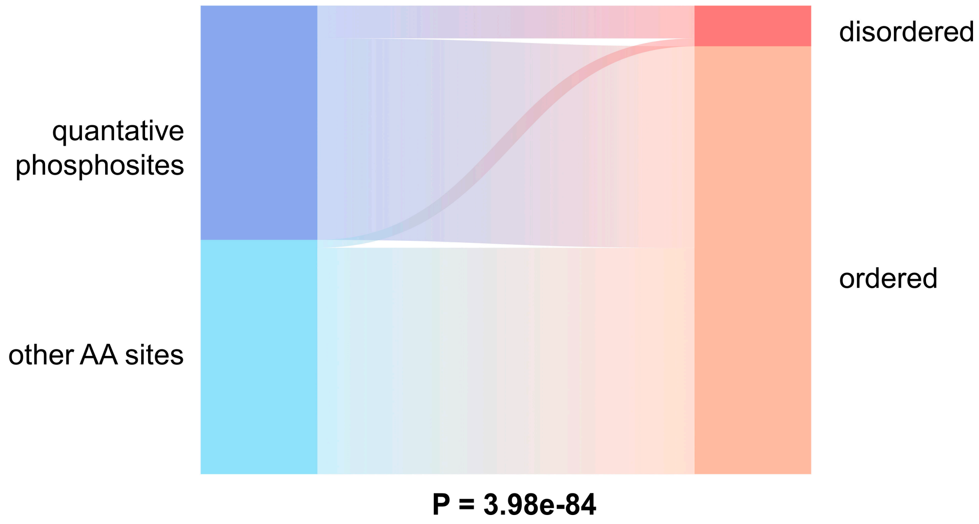

Supplement: Supplementary file 1 [file ijms-24-14817-s001.zip › Supplementary Figures.pdf]
